# Determination of the Modulatory Effects of Selenium-Enriched Egg Powder on the Physiological Immune Response and Cecal Microbiota of Kunming Mice

**DOI:** 10.3390/foods15061069

**Published:** 2026-03-18

**Authors:** Min Xie, Fuguang Xue, Mengjie Sun, Qinghao Zhuang, Shiyi Tang, Yuxin Huang, Yao Zhang, Jingyi Hu, Yaomin Zhou

**Affiliations:** 1 Institute of Quality and Safety of Agricultural Products and Standards, Jiangxi Academy of Agricultural Sciences, Nanchang 330200, China; zbsxiemin@163.com; 2 Nanchang Key Laboratory of Animal Health and Safety Production, Jiangxi Agricultural University, Nanchang 330045, China; xuefuguang123@163.com (F.X.); 17629316589@163.com (M.S.); 15557808771@163.com (Q.Z.); 19162989806@163.com (S.T.); m17879017026_2@163.com (Y.H.); 17870913556@139.com (Y.Z.); 18379160751@163.com (J.H.)

**Keywords:** se-enriched eggs, mice, gut microbiota, growth performance, immunity

## Abstract

Se-enriched functional eggs are prevalent nowadays, which may help improve body health and anti-oxidant capacities. However, the modulatory effects on cecal microbiota are still limited. This study aims to investigate the underlying mechanism of Se-enriched egg powder in modulating the cecal microbiota of Kunming mice. A total of 72 mice were randomly assigned to a control treatment (CON), a conventional egg powder treatment (EP), and four gradient Se-enriched egg powder treatments (EPS1–EPS4, with the Se content ranging from 0.01 to 0.04% of total dietary content) for a 35-day feeding procedure. Parameters included growth performance, tissue Se content distribution, serum anti-oxidant capacities (GSH-Px, SOD, MDA), and immune cytokines (IgG, TNF-α), and cecal microbiota composition was further measured. Results showed dietary 0.02% (EPS2) significantly improved growth performance, physiological anti-oxidant defenses, and cytokine TNF-α (*p* < 0.05), while significantly reducing feed conversion ratio and malondialdehyde (MDA) compared with CON (*p* < 0.05). Metagenomic results revealed that Se-enriched egg powder significantly increased bacterial α-diversity and the abundance of *Akkermansia*, *Bacteroides*, and *Bifidobacterium* (*p* < 0.05), while significantly decreasing *Desulfovibrio* and *Escherichia-Shigella* (*p* < 0.05). In conclusion, dietary supplementation with Se-enriched egg powder effectively enhances growth performance, anti-oxidant capacity, and immunity, mainly through the promotion of beneficial bacteria diversity and suppression of pathogens.

## 1. Introduction

Selenium (Se), mainly worked through selenoproteins, plays a pivotal physiological role in human processes, including the anti-oxidant defense, immune function, thyroid hormone metabolism, DNA synthesis, and reproduction [1,2,3,4]. Research focused on Se metabolism showed the significant anti-oxidant capacities of selenium-dependent enzymes like glutathione peroxidases (GPx) [5] and thioredoxin reductases (TrxR) [6] by reducing harmful peroxides, and the increased viral susceptibility and potential cancer risk induced by Se deficiency [5]. The Recommended Dietary Allowance (RDA) is 55 µg/day, with a tolerable upper limit of 400 µg/day [3]. However, average daily Se acquisition of the majority was seriously inadequate compared to the recommendation, with less than 30 µg [4]; therefore, a proper Se supplementation method was urgently needed to help improve daily Se acquisition.

Biofortification was considered an effective pathway to improve daily Se acquisition, of which the selenium-enriched eggs became an effective functional food in alleviating selenium deficiency. Laying hens fed with supplementation of organic selenium, such as selenomethionine or selenium-enriched yeast [7], were proven to have 3–10 times higher selenium content than conventional eggs due to superior bioavailability [8,9]. Selenium-enriched eggs offered a convenient dietary Se source, easy acquisition, and lower prices that help enhance daily Se acquisition [10]. Additionally, the anti-oxidant capacities and relative abundances of cecal microbiota were also significantly enhanced after receiving Se-enriched eggs [11,12]. However, the optimal daily Se-enriched egg intake and the modulatory mechanism on microbiota, physiological immunity, and interactive effects with nutrient metabolism and redox balance are not well characterized.

Therefore, this study aims to address the optimum dosage of Se-enriched eggs and downstream modulatory effects on gastrointestinal microbiota, physiological anti-oxidant capacity, and immunity. Model organisms of Kunming mice were selected to investigate dose–response effects and tissue-specific analyses because of the easy acquisition, lower feeding cost, and high genomic homology with human beings. We hypothesize that Se-enriched eggs enhance growth performance and systemic anti-oxidant capacity through modulating gastrointestinal microbiota, enhancing nutrient digestibility, and improving nutritional efficiency.

## 2. Materials and Methods

### 2.1. Ethical Statement

Animal care and experimental procedures were conducted in accordance with the Chinese Guidelines for Animal Welfare. The study was approved by the Animal Care and Use Committee of Jiangxi Agricultural University (Approval No. JXAULL-20250226).

### 2.2. Se-Enriched Egg Acquirement and Egg Powder Preparation

A total of 200 healthy Lohmann Pink laying hens aged at 305 days with similar body weights (1.95 ± 0.17 kg) were randomly divided into two treatments, the control group and the selenium-enriched group. Each group contains 10 replicates, with 10 hens per replicate. The control group was fed with a basal diet, while the selenium-enriched group was fed with a basal diet supplemented with 0.5% selenium-enriched nutrients. All hens in each treatment were reared in the same stall, and provided with a restricted 110 g of feedstuff at 8:00 a.m. per day.

The selenium-enriched nutrients, mainly including the corn and soybean meal, were sourced from the core area of the Ecological Selenium Valley in Fengcheng, Jiangxi Province. The compound probiotic culture was provided by Guangdong Lishengyuan Biological Feed Co., Ltd. (Guangzhou, China). (containing *Saccharomyces cerevisiae*, *Lactobacillus*, and *Bacillus subtilis*, with a total probiotic count ≥ 3 × 10^9^ CFU/g). The selenium-enriched nutrients were anaerobically fermented in a 36 °C constant-temperature incubator for 7 days. The final product contained 0.448 mg/kg selenium.

At the end of treatment, ten eggs from each replicate were selected for the Se-content measurement. All eggs were receiving a freeze-drying process in the lyophilizer (YTLG-10A, Shanghai Yeto Co., Ltd., Shanghai, China). The Se content of egg powder was measured based on the National Standard of Se Content Measurement (GB5009.93-2017) [13], which includes the pretreatment, the hydrogenide preparation, and the Atomic Fluorescence Spectroscopy measurement of hydrogenide.

### 2.3. Se-Enriched Diet for Mice Preparation and Experimental Design

The Se-enriched diet for mice treatment was set according to the recommendation of daily Se acquisition for human beings (50–250 μg/day, and the maximum daily intake is 400 μg/day). The daily Se acquisition for mice was equivalent to a transformation based on the body weight, with the average body weight set at 30 g for mice during the process and the body weight at 60 kg for human beings. The recommended daily Se intake for mice was calculated through the following equation:

Se_(mice)_ = Se_(__human)_ × (average BW_(mice)_/average BW_(human)_)

where BW stands for the body weight. Therefore, the recommendation of daily Se acquisition for mice was 0.025 μg/day to 0.125 μg/day, and the maximum daily intake was 0.2 μg/day. Daily feed intake for each mouse was considered to be 6 g/day based on previous studies. All ingredients and chemical compositions for each treatment are displayed in Table 1.

A total of 72 three-week-old Kunming (KM) male mice with an average body weight of 19.1 ± 0.3 g were acquired from Hunan SJA Laboratory Animal Co., Ltd. (Changsha, China, http://www.hnsja.com/index.html, accessed on 7 March 2025). All mice were randomly divided into the control treatment (CON, fed with standard chow), the egg powder supplement treatment (EP), and the gradient increasing Se-enriched egg powder (EPS) treatments. Each treatment contained 6 repeats, with 2 mice in each repeat. All animals were housed in stand cages (*n* = 2 mice per cage) in the same room, exposed to the environment with 22 ± 2.0 °C of temperature, 45 ± 5.0% of humidity, and a 12 h light/dark cycle lighting program. Mice were acclimatized for a 35-day-long experimental process, and given free access to standard chow and water.

### 2.4. Parameters Measurement

#### 2.4.1. Growth Performance

The body weight (BW) of each mouse at the initial and final stages was measured based on the unit of replicate to calculate the body weight gain (BWG) and average daily weight gain (ADG). Feed intake (FI) was calculated by the deviation between the initial feed supply and the end feed residue at the end of each week. Feed conversion ratio (FCR) was calculated using the following equation.

FCR = FI(g)/BWG(g).

#### 2.4.2. Blood and Organ Se Content Measurement

Blood samples of each mouse were collected at the end of the trial through the orbital blood collection method, and a total of 100 μL of blood sample was separated for the Se-content measurement. In addition, samples of skin, liver, and thigh muscle were collected from each mouse, and all samples were applied for Se-content measurement. Methods used for Se-content measurement were the same as the above-mentioned.

#### 2.4.3. Blood Anti-Oxidant and Immunity-Related Parameters Measurement

A total of 300 μL blood samples from each mouse were collected at the end of the trial through the orbital blood collection method. Anti-oxidant-related parameters, including the superoxide dismutase (SOD), glutathione peroxidase (GSH-px), and malondialdehyde (MDA), and immunity-related parameters, including the tumor necrosis factor-α (TNF-α), and immune globulins such as IgA, IgM, IgE, and IgG, were determined to investigate the Se-enriched egg powder on physiological anti-oxidant capacity and immunity.

SOD and GSH-px were measured using spectrophotometric assays by the enzyme-linked immunosorbent assay (ELISA) detection method provided by Nanjing Jiancheng Biotech Company (Nanjing, China). Absorbance at 340 nm was measured, and the activity was calculated through the regression equation provided by the instructions of the toolkit. The tumor necrosis factor-α (TNF-α) and immune globulins such as IgA, IgM, and IgG were measured through ELISA methods, which were provided by Nanjing Jiancheng Biotech Company and quantified using the AU5421 Automatic Biochemistry Analyzer (Backman Coulter, Inc., Brea, CA, USA) at the First Affiliated Hospital of Nanchang University.

#### 2.4.4. Cecal Microbiota Measurement

A total of 0.5 g of cecal samples of each replicate were collected at the end of the trial after mice were sacrificed, and samples were rapidly frozen by liquid nitrogen and stored at −80 °C for 16S rRNA sequencing analysis. Methods used for 16S rRNA bacterial diversity analysis were the same as in our previous study [14]. Briefly, DNA samples were extracted, followed by the concentration and purity measurement. The V4 region of 16S rRNA was amplified using the universal primers 520F and 802R (F: GTGCCAGCMGCCGCGGTAA and R: GGACTACHVGGGTWTCTAAT) for the PCR amplification process. Sequencing libraries were established using the TruSeq^®^ DNA PCR-Free Sample Preparation Kit (Illumina Inc., San Diego, CA, USA), followed by the sequencing process using the Illumina HiSeq 4000 platform (Illumina Inc., San Diego, CA, USA). Quantitative Insights Into Microbial Ecology (QIIME, V2.0) [15] was applied for the quality control process, followed by the application of the SILVA classifier algorithm (www.arb-silva.de) to annotate taxonomic information. The effective tags were obtained and analyzed by Uparse software (Uparse v7.0.1001), and sequences with similarity > 97% were assigned to the same operational taxonomic unit (OTU) and applied for microbial community identification. Relative abundances of cecal bacteria after filtration were normalized first to fit the standard of differential analysis, and further received the α-diversity and β-diversity analysis. Principal coordinate analysis (PCoA) for bacterial communities was conducted using the R package version 3.3.1 (R Core Team, Vienna, Austria). A functional prediction process was conducted using the Tax4Fun method based on the significantly different microbiota [16].

### 2.5. Statistical Analysis

A normal distribution test on growth performances, serum immunity-related parameters, anti-oxidant capacities of each treatment was first conducted using the SAS 9.2 (SAS Institute Inc., Cary, NC, USA) procedure “proc univariate data = test normal”, and the normality was followed by a test using the SAS procedure “Shapiro–Wilk”. The one-way ANOVA S-N-K test, which used dynamically adjusted critical values during the step-by-step comparison process, was applied to investigate the differences (SAS Institute Inc., Cary, NC, USA). Results were presented as means ± SEM, and a *p*-value < 0.05 was considered significant, and 0.05 ≤ *p* < 0.10 was considered as a tendency.

## 3. Results

### 3.1. Effects of Se-Enriched Egg Powder on Growth Performances of Kunming Mice

Growth performances, including body weight, body weight gain, feed intake, and FCR, were detected, and the results are shown in Table 2. The final body weight, body weight gain, and ADG of mice in EPS2 and EPS3 treatments were significantly higher than CON, EP, EPS1, and EPS4 treatments (*p* < 0.05). However, no significant alterations were observed for FI and average daily feed intake (ADFI) among all groups (*p* > 0.05). FCR in EPS2 and EPS3 treatments is significantly lower than that in CON, EP, EPS1, and EPS4 treatments (*p* < 0.05).

### 3.2. Serum Anti-Oxidant Capacity and Immunity-Related Parameters

Effects of Se-enriched egg powder supplement on serum anti-oxidant parameters are shown in Table 3. The content of GSH-px significantly increased in EPS2 and EPS3 treatments compared with the other four treatments (*p* < 0.05), while MDA content in EPS2 and EPS3 treatments was significantly lower than that in CON (*p* < 0.05). SOD content in the EPS2 treatment significantly increased compared with CON, EP, and EPS4 treatments (*p* < 0.05). Ig G content in EPS2 and CON was significantly lower than that in EPS4 treatment (*p* < 0.05). Meanwhile, the TNF-α content in EPS2 and EPS3 treatments was significantly lower than that in CON, EP, and EPS4 treatments (*p* < 0.05). No significant alterations were found for IgM and IgA content among all treatments.

### 3.3. Selenium Content Measurement in Different Body Organs

Effects of the Se-enriched egg powder supplement on the selenium content in different body organs were shown in Table 4. Selenium content in different organs showed a significant positive correlation with dietary selenium supply, and selenium content in EPS2, EPS3, and EPS4 treatments showed significant increments compared with CON, EP, and EPS1 treatments in muscle, liver, and skin organs (*p* < 0.05). Blood selenium content in EPS3 and EPS4 treatments showed significant increments compared with CON and EP treatments (*p* < 0.05). No significant alterations of selenium content were found in blood between EPS1 and EPS2 treatments (*p* > 0.05), while no significant changes were found between CON and EP treatments in muscle, liver, blood, and skin (*p* > 0.05).

### 3.4. Effect of Se-Enriched Egg Powder on Cecal Microbiota of Kunming Mice

EPS2 treatment was chosen as the optimum Se-enriched egg powder replacement dosage based on the results of growth performances, anti-oxidant capacities, immunity, and selenium content stated above. The cecal microbiota was further investigated to determine the effect of Se-enriched egg powder on Kunming mice in the CON, EP, and optimum EPS treatments. A total of 12 phyla and more than 180 genera were identified based on the filtration method, and all the bacterial communities are shown in Supplementary Materials Table S1. All communities are selected for further α-diversity and β-diversity analysis.

#### 3.4.1. α-Diversity

Alpha diversity was displayed through Chao1, Shannon, Simpson, ACE, Pielou, PD, and goods_coverage indexes, and shown in Table 5. Indexes of Chao1, ACE, Shannon, Pielou, and PD in EP and EPS treatments significantly increased compared with CON (*p* < 0.05). No significant alterations were detected on Simpson, and goods_coverage indexes after egg powder and Se-enriched egg powder replacement compared with CON (*p* > 0.05).

#### 3.4.2. β-Diversity

**Figure 1:**
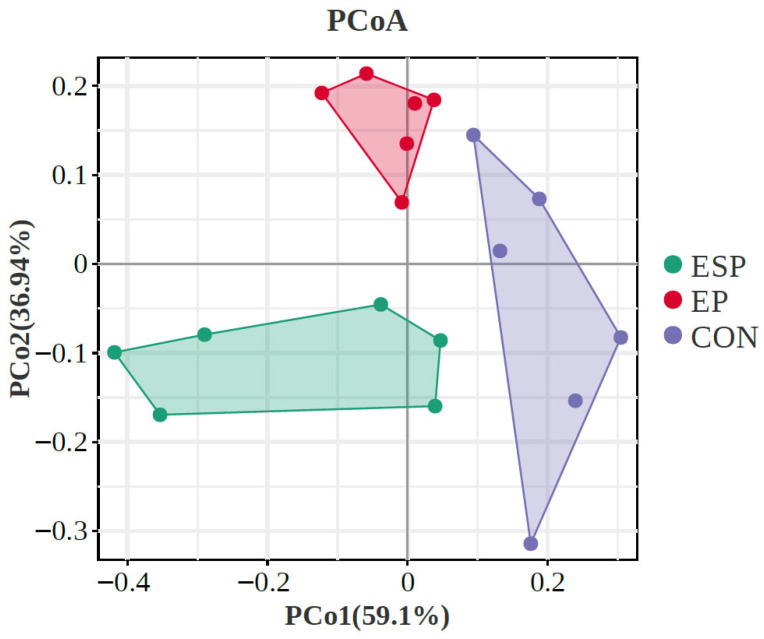
Principal coordinate analysis (PCoA) on community structures of the gut microbiota of CON, Milk, and KPM treatments. CON = control treatment; EP = egg powder; EPS = Se-enriched egg powder.

Principal coordinates analysis (PCoA) focused on clarifying the monolithic discrepancy of bacterial profiles among all treatments, as shown in Figure 1. PCoA axes 1 and 2 accounted for the integrative interpretation of 59.10% and 36.94%, respectively. Bacterial communities in EP and EPS treatments were clearly separated from those in CON through PCoA axis 1, while bacterial communities in EP treatment were significantly separated from those in EPS treatment.

Differential analysis on the relative bacterial abundances at both phyla and genus levels was conducted to investigate the effect of EPS supplement on cecal microbial communities, and is shown in Table 6 and Table 7. *Firmicutes*, *Bacteroidota*, and *Desulfobacterota* contributed the largest populations of whole bacteria, and accounted for more than 90% of the total microbiota. The relative abundance of *Firmicutes* significantly decreased in EPS treatment compared with EP (*p* < 0.05), while *Bacteroidota* significantly increased in EPS compared with EP and CON (*p* < 0.05). The relative abundance of *Desulfobacterota* significantly decreased in both EP and EPS treatments compared with CON. *Patescibacteria* significantly decreased after receiving selenium-enriched egg powder treatment compared with CON and EP treatments (*p* < 0.05). No other significant changes were found in other phyla among all three treatments.

In the genera level, *Bacteroides*, *Muribaculaceae*, *Akkermansia*, *Desulfovibrio*, *Corynebacterium*, and *Oligella* accounted for the top 6 genera of all three treatments. *Bacteroides*, *Akkermansia*, *Parasutterella*, and *Bifidobacterium* significantly increased after receiving Se-enriched egg powder treatment compared with CON and EP treatments (*p* < 0.05). Relative abundance of *Lactobacillus* significantly proliferated, while Desulfovibrio and Escherichia-Shigella significantly decreased in EP and EPS treatments compared with CON (*p* < 0.05). No significant changes were investigated for other genera (*p* > 0.05).

**Figure 2:**
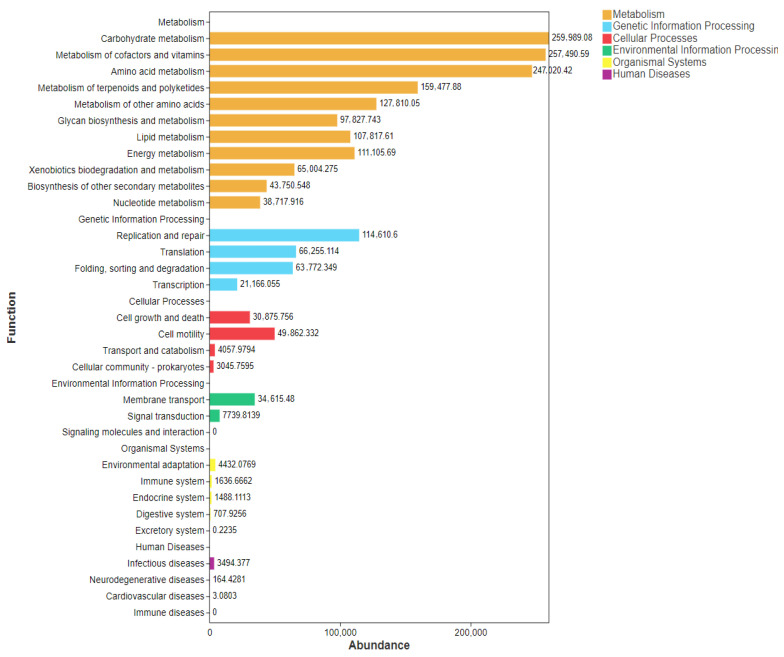
Functional prediction analysis of the relative abundances of bacterial communities using Tax4Fun.

Functional prediction results based on the relative abundances of microbial communities are shown in Figure 2. Metabolism, genetic information processing, and cellular processes are the main enriched predictive functions for the microbial communities in the different microbial communities. Metabolic processes, including carbohydrate metabolism, metabolism of cofactors and vitamins, and amino acid metabolism, are detected as the predominant functional pathways that are affected by selenium enrichment. Additionally, the genetic information processing processes mainly include the replication, repair, and translation programs. Cell growth and cell motility are the main enriched cellular processes in response to selenium enrichment.

### 3.5. Correlation Analysis Between Growth Performances, Anti-Oxidant Capacities, Immunity, and Cecal Microbiota

**Figure 3:**
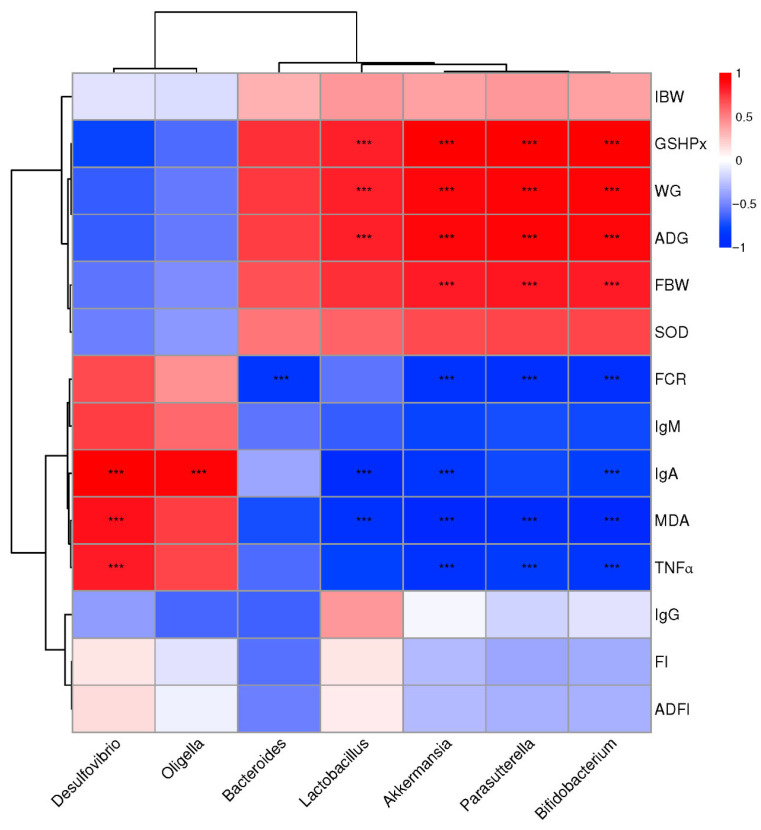
Correlation analysis between abundances of cecal bacteria and mice production performances, intestinal development parameters, and blood lipid parameters at the level of genera. The red color represents a positive correlation, while the blue color represents a negative correlation. “***” means a significant correlation (|r| > 0.55, *p* < 0.05). FI = feed intake, WG = weight gain, ADG = average daily weight gain, FBW = final body weight, FCR = feed conversion ratio, GSH-PX = glutathione peroxidase, SOD = superoxide dismutase, MDA = malondialdehyde.

Correlation analysis between the significantly altered bacterial communities after Se-enriched egg powder supplement and growth performances, anti-oxidant capacities, and immunity-related parameters was conducted, and the result is shown in Figure 3.

Integrally, the phenotypic parameters were separated into two big clusters. One was mainly composed of GSH-px, WG, ADG, FBW, and SOD, which showed a positive correlation with *Bacteroides*, *Akkermansia*, *Parasutterella*, *Lactobacillus*, and *Bifidobacterium*, while negatively correlated with *Desulfovibrio* and *Oligella*. The other part mainly consisted of FCR, IgM, MDA, IgA, and TNF-α, which showed a converse correlation with the above-mentioned bacterial communities compared with the first cluster.

Specifically, parameters of GSH-px, WG, ADG, and FBW showed a significant correlation with *Akkermansia*, *Parasutterella*, and *Bifidobacterium*, while GSH-px, WG, and ADG showed a significant correlation with *Lactobacillus*. FCR showed a significant negative correlation with *Bacteroides*, *Akkermansia*, *Parasutterella*, and *Bifidobacterium*. The content of MDA, IgA, and TNF-α showed a significant positive correlation with Desulfovibrio, while it was significantly negatively correlated with *Akkermansia*, *Parasutterella*, *Lactobacillus*, and *Bifidobacterium*. Particularly, *Oligella* are significantly correlated with IgA while showing no significant correlation with other phenotypic parameters. No other significant correlations between phenotypic parameters and bacterial communities were detected.

## 4. Discussion

Dietary replacement with an optimal dosage of Se-enriched egg powder significantly improved growth performance, enhanced the anti-oxidant defense system, and suppressed pro-inflammatory cytokine secretion [12,16,17], which were mainly achieved by remodeling gut microbiota structure, increasing the relative abundances of beneficial bacteria such as *Akkermansia* and *Bifidobacterium* [18,19], and inhibiting potential pathogens. Our findings supported the potential application of Se-enriched egg powder as a functional food additive, and might work through a mechanism of “Dietary Selenium-Gut Microbiota-Host Health” axis.

Growth performance was primarily investigated to evaluate the efficacy of Se-enriched egg powder. Our findings indicated that mice that received Se supplements exhibited a higher final body weight and higher ADG, and a lower FCR compared to the control and conventional egg powder groups. Our findings displayed a similar result to Liu [20], and suggest nutritional enhancement and growth promotion effects of Se-enriched egg powder. Reasons could be mainly attributed to the high absorptivity and bioavailability of organic selenium that exists in the Se-enriched egg powder [21]. The absorbed selenium participated in the synthesis of body proteins and further actively promoted the conversion of thyroxine (T4) to the biologically active triiodothyronine (T3) [17], which subsequently triggered the regulatory function in basal metabolic protein synthesis. Thereby, the growth performances were significantly enhanced after the Se-enriched egg powder supplement. Furthermore, the improved feed efficiency is likely associated with an altered gastrointestinal microbiome. As our results showed, optimal Se-enriched egg powder treatment significantly improved bacterial α-diversity, indicated a higher diversity of bacterial communities, a stable and resilient gut ecosystem, and robust degradability of nutritional elements. Specifically, *Akkermansia muciniphila* was well detected for degrading mucin to maintain gut barrier integrity and is closely associated with improved metabolic health [19,22], while *Lactobacillus* and *Bifidobacterium* specialized in breaking down complex polysaccharides to produce lactate and acetate [18,23], which significantly improved host energy metabolism, and therefore promoted body growth. Conversely, Se-enriched egg powder significantly reduced the abundance of *Desulfovibrio* [24,25], and *Escherichia-Shigella* [26,27] and restricted the production of toxic secondary metabolites such as hydrogen sulfide (H_2_S) to break the integrity of intestinal epithelial cells. Therefore, dietary nutrients could be more efficiently degraded, and partially interpreted the increased feed efficiency. Moreover, Spearman correlation analysis further confirmed the strong link between microbial shifts and growth performance. This suggests that Se-enriched egg powder suppresses endotoxin-producing or metabolically harmful bacteria while promoting SCFA-producing bacteria, ultimately fostering growth [4]. As our results showed, a significant enrichment of probiotics, including the *Akkermansia*, *Lactobacillus*, and *Bifidobacterium*, and inhibition of pathogens such as the *Desulfovibrio* and *Escherichia-Shigella,* further confirmed the linkage.

Improvement of body health was a well-proven effect of Se treatment, which was also found in our present study by selenium-enriched egg powder treatment. The primary physiological function of selenium is exerted through the anti-oxidant activity of selenoproteins, which indicates a powerful anti-oxidant capacity of selenium enrichment, reflected by the significantly elevated serum GSH-Px and SOD activities in the EPS treatment, while the significantly reductive content of malondialdehyde (MDA), a lipid peroxidation marker [21]. GSH-Px acts as the body’s first line of defense by scavenging hydrogen peroxide (H_2_O_2_) and lipid hydroperoxides. The intake of Se-enriched egg powder directly provides the selenium required for GSH-Px synthesis, and upregulates the enzymatic activity to resist an oxidative environment. As previous research showed, the increased anti-oxidant ability after receiving Se-enriched powder treatment protects metabolically active organs to maintain efficient anabolic functions, such as the liver and muscle, and reduces the energy expenditure required for repairing oxidative damage [1,12,22], thereby reallocating more energy towards growth, which aligns with our findings on growth performance. EPS treatment showed significantly reduced levels of the pro-inflammatory cytokine TNFα [28]. Reasons might contribute to the significantly improved anti-oxidant capacities of Se-enriched egg powder, which may activate NF-κB inflammatory pathways [29] and suppress the excessive secretion of TNF-α [30].

## 5. Conclusions

**Figure 4:**
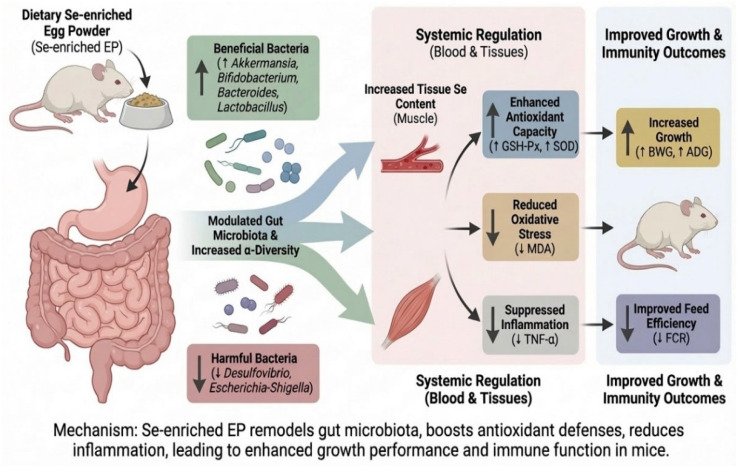
Mechanism of Se-enriched egg powder in modulating growth performance and immunity.

In summary, this study demonstrates that dietary supplementation with an appropriate dosage of Se-enriched egg powder significantly enhances the growth performance of Kunming mice. The underlying mechanism is multidimensional, as Figure 4 shows: Se-enriched egg powder provides organic selenium to boost anti-oxidant enzyme activities (GSH-Px, SOD) and suppress excessive inflammation (lower TNF-α), as well as an indirect pathway of remodeling the gut micro-ecology. Specifically, the treatment enriched beneficial genera such as *Akkermansia* and suppressed harmful genera like Desulfovibrio, thereby improving gut health and metabolic efficiency. These findings provide a scientific basis for developing Se-enriched egg powder as a functional food capable of simultaneously targeting oxidative stress and gut microbiota. Future studies should integrate metabolomics to further elucidate the specific signaling mechanisms of microbial metabolites in this process.

## Figures and Tables

**Table 1:** Ingredients and chemical composition of all treatment diets (dry matter basis).

Items (%)	CON	EP	EPS1	EPS2	EPS3	EPS4
Ground Corn	39	39	39	39	39	39
Wheat bran	20	20	20	20	20	20
Wheat flour	15	15	15	15	15	15
Soybean meal	17	16	16.4	15.4	14.4	12.4
Egg powder (EP)	0	3				
Se-enriched EP			1.6	3.6	5.6	7.6
Fish meal	4	2	3	2	1	1
NaCl	0.5	0.5	0.5	0.5	0.5	0.5
Limestone	1.5	1.5	1.5	1.5	1.5	1.5
CaHPO_4_	2	2	2	2	2	2
Premix ^(^^1)^	1	1	1	1	1	1
Nutritional levels (%)						
GE (MJ/kg)	14.21	14.23	14.21	14.23	14.25	14.27
EE	4.50	4.56	4.51	4.56	4.59	4.61
CP	18.1	18.1	18.1	18.1	18.1	18.1
Se	0.002	0.005	0.01	0.02	0.03	0.04
Ca	1.2	1.2	1.2	1.2	1.2	1.2
P	0.8	0.8	0.8	0.8	0.8	0.8

^(1)^. The components contained in the premix are as follows: Fe, 100 mg; Cu, 12 mg; Mn, 75 mg; Zn, 30 mg; I, 0.5 mg; VA, 900,000 IU; VB1, 8 mg; VB2, 10 mg; VB6, 6 mg; VD, 800 IU; and VE, 60 IU. CON = control treatment, EP = egg powder replacement treatment. EPS = se-enriched egg powder replacement treatment. The Se content of common eggs was measured at 0.1 mg/kg, while the Se-enriched eggs were measured at 0.5 mg/kg. Se was removed from the premix.

**Table 2:** Effects of Se-enriched egg powder supplement on the growth performances of Kunming mice (*n* = 6).

Items	CON	EP	EPS1	EPS2	EPS3	EPS4	SEM	*p*-Value
Initial body weight (g)	19.33	19.37	19.17	18.92	19.72	19.47	1.02	0.643
Final body weight (g)	43.03 ^b^	43.8 ^b^	43.81 ^b^	45.8 ^a^	46.38 ^a^	44.17 ^b^	0.97	0.032
Body weight gain (g)	23.70 ^b^	24.42 ^b^	24.64 ^b^	26.57 ^a^	26.66 ^a^	24.7 ^b^	0.48	0.017
ADG (g)	0.68 ^b^	0.70 ^b^	0.70 ^b^	0.76 ^a^	0.76 ^a^	0.71 ^b^	0.03	0.041
FI (g)	196.4	202.5	201.1	197	193.1	198.4	7.64	0.124
ADFI (g)	5.61	5.74	5.73	5.63	5.52	5.67	0.32	0.486
FCR	8.29 ^a^	8.25 ^a^	8.21 ^a^	7.41 ^b^	7.24 ^b^	8.03 ^a^	0.13	0.039

^a,b^ means a significant difference was detected in the same row among treatments. ADG = average daily weight gain, FI = feed intake, ADFI = average daily feed intake, and FCR = feed conversion ratio. CON = control treatment, EP = egg powder, EPS = Se-enriched egg powder, and SEM = standard error of mean.

**Table 3:** Effects of Se-enriched egg powder supplement on the anti-oxidant capacities and immunity-related parameters of Kunming mice (*n* = 6).

Items	CON	EP	EPS1	EPS2	EPS3	EPS4	SEM	*p*-Value
GSH-Px (U/mL)	126.1 ^b^	129.3 ^b^	132.6 ^b^	138.4 ^a^	141.3 ^a^	131.2 ^b^	2.49	0.026
SOD (U/mL)	612.3 ^b^	616.3 ^b^	621.4 ^ab^	629.3 ^a^	622.8 ^ab^	611.2 ^b^	11.30	0.042
MDA (μM)	2.83 ^a^	2.64 ^ab^	2.53 ^ab^	2.32 ^b^	2.41 ^b^	2.48 ^ab^	0.15	0.039
IgA (g/L)	2.00	1.83	1.82	1.78	1.85	1.89	0.17	0.131
IgG (g/L)	17.21 ^b^	18.45 ^ab^	18.37 ^ab^	17.41 ^b^	18.43 ^ab^	19.48 ^a^	0.28	0.095
IgM (g/L)	1.39	1.37	1.35	1.34	1.31	1.36	0.05	0.182
TNF-α (pg/mL)	62.92 ^a^	61.32 ^a^	60.34 ^ab^	59.37 ^b^	58.31 ^b^	63.28 ^a^	1.02	0.031

^a,b^ means a significant difference was detected in the same row among treatments. CON = control treatment, EP = egg powder, EPS = Se-enriched egg powder, SEM = standard error of mean. GSH-PX = glutathione peroxidase, SOD = superoxide dismutase, MDA = malondialdehyde.

**Table 4:** Effects of Se-enriched egg powder supplement on the Se content in different sections of Kunming mice (*n* = 6).

Items	CON	EP	EPS1	EPS2	EPS3	EPS4	SEM	*p*-Value
Muscle (mg/kg)	0.16 ^b^	0.19 ^b^	0.21 ^b^	0.24 ^a^	0.27 ^a^	0.28 ^a^	0.03	0.013
Blood (mg/L)	0.07 ^b^	0.08 ^b^	0.11 ^ab^	0.13 ^ab^	0.16 ^a^	0.18 ^a^	0.01	0.001
Liver (mg/kg)	0.14 ^b^	0.16 ^b^	0.19 ^b^	0.23 ^a^	0.26 ^a^	0.26 ^a^	0.04	0.026
Skin (mg/kg)	0.07 ^b^	0.08 ^b^	0.09 ^b^	0.11 ^a^	0.13 ^a^	0.14 ^a^	0.02	0.011
Se absorption (%)	74.23	75.26	76.11	77.32	76.74	75.64	2.41	0.247

^a,b^ means a significant difference was detected in the same row among treatments. CON = control treatment, EP = egg powder, EPS = Se-enriched egg powder, and SEM = standard error of mean.

**Table 5:** Effects of Se-enriched egg powder supplement on the α-diversity of fecal microbiota of Kunming mice (*n* = 6).

Items	CON	EP	EPS	SEM	*p*-Value
Chao1	68.43 ^b^	75.17 ^a^	79.50 ^a^	2.36	<0.001
ACE	68.43 ^b^	75.17 ^a^	79.58 ^a^	2.37	<0.001
Shannon	1.94 ^b^	2.42 ^a^	2.56 ^a^	0.10	0.008
Simpson	0.80	0.80	0.84	0.03	0.275
Pielou	0.48 ^b^	0.56 ^a^	0.58 ^a^	0.02	0.045
goods_coverage	1.00	1.00	1.00	0.01	0.516
PD	7.75 ^b^	8.99 ^a^	9.35 ^a^	0.19	0.037

^a,b^ means a significant difference was detected in the same row among treatments. CON = control treatment, EP = egg powder, EPS = Se-enriched egg powder, and SEM = standard error of mean.

**Table 6:** Effects of Se-enriched egg powder supplement on fecal microbiota of Kunming mice (level of phyla, *n* = 6, %).

Items	CON	EP	EPS	SEM	*p*-Value
*p__Firmicutes*	35.82 ^ab^	38.65 ^a^	32.93 ^b^	3.57	0.011
*p__Bacteroidota*	29.16 ^b^	28.31 ^b^	37.35 ^a^	4.54	0.021
*p__Desulfobacterota*	30.75 ^a^	25.25 ^b^	24.66 ^b^	4.18	0.003
*p__Actinobacteriota*	2.52	4.69	2.07	0.70	0.245
*p__Proteobacteria*	0.40	0.91	1.62	0.17	0.116
*p__Campilobacterota*	0.34	0.09	0.69	0.23	0.483
*p__Patescibacteria*	0.06 ^a^	0.08 ^a^	0.02 ^b^	0.00	0.002
*p__Verrucomicrobiota*	0.70	1.87	0.19	0.41	0.193

^a,b^ means a significant difference was detected in the same row among treatments. CON = control treatment, EP = egg powder, EPS = Se-enriched egg powder, SEM = standard error of mean.

**Table 7:** Effects of Se-enriched egg powder supplement on fecal microbiota of Kunming mice (level of genera, *n* = 6, %).

Items	CON	EP	EPS	SEM	*p*-Value
*g__Bacteroides*	24.33 ^b^	22.74 ^b^	27.13 ^a^	2.95	0.037
*g__Muribaculaceae*	18.17	22.70	20.03	4.74	0.343
*g__Akkermansia*	0.22 ^b^	2.50 ^b^	7.07 ^a^	1.92	0.013
*g__Desulfovibrio*	5.70 ^a^	3.54 ^b^	2.97 ^b^	0.83	0.021
*g__Corynebacterium*	3.21	2.18	2.15	0.71	0.805
*g__Oligella*	5.18 ^a^	0.32 ^b^	0.73 ^b^	0.60	0.012
*g__Alistipes*	2.32	2.06	0.75	0.40	0.238
*g__Colidextribacter*	1.91	1.63	0.48	0.30	0.112
*g__Alloprevotella*	0.41	2.10	1.54	0.41	0.233
*g__Staphylococcus*	2.71	0.74	0.81	0.51	0.202
*g__Lactobacillus*	1.32 ^b^	2.02 ^a^	2.23 ^a^	0.37	0.016
*g__Helicobacter*	0.31	0.11	1.88	0.60	0.443
*g__Roseburia*	0.37	0.89	0.97	0.29	0.687
*g__Enterorhabdus*	0.88	0.72	0.60	0.22	0.891
*g__Rikenella*	0.25	1.50	0.07	0.42	0.350
*g__Odoribacter*	0.99	0.28	0.36	0.16	0.127
*g__Monoglobus*	0.26	0.58	0.27	0.14	0.608
*g__Parabacteroides*	0.32	0.31	0.38	0.06	0.870
*g__Streptococcus*	0.24	0.61	0.17	0.10	0.174
*g__Negativibacillus*	0.15	0.04	0.14	0.03	0.179
*g__Romboutsia*	0.06	0.22	0.18	0.06	0.583
*g__Parasutterella*	0.05 ^b^	0.07 ^b^	0.17 ^a^	0.03	0.017
*g__Ruminococcus*	0.14	0.02	0.16	0.04	0.275
*g__Atopostipes*	0.10	0.07	0.15	0.03	0.663
*g__Oscillibacter*	0.05	0.02	0.03	0.01	0.585
*g__Butyricicoccus*	0.02	0.08	0.04	0.02	0.292
*g__Bifidobacterium*	0.01 ^b^	0.04 ^b^	0.13 ^a^	0.02	0.003
*g__Sellimonas*	0.03	0.03	0.05	0.01	0.250
*g__Acetitomaculum*	0.05	0.01	0.02	0.01	0.222
*g__Escherichia-Shigella*	0.04 ^a^	0.01 ^b^	0.01 ^b^	0.00	0.005
others	30.21	29.81	28.31	2.40	0.063

^a,b^ means a significant difference was detected in the same row among treatments. CON = control treatment, EP = egg powder, EPS = Se-enriched egg powder, SEM = standard error of mean.

## Data Availability

The original contributions presented in this study are included in the article/Supplementary Material. Further inquiries can be directed to the corresponding author.

## Supplementary Materials

The following supporting information can be downloaded at https://www.mdpi.com/article/10.3390/foods15061069/s1, Table S1: Microbial communities of the CON, EP, and EPS treatments in mice.

## Abbreviations

The following abbreviations are used in this manuscript:

EP	Egg Powder
EPS	Se-enriched Egg Powder
GPx	Glutathione Peroxidases
TrxR	Thioredoxin Reductases
RDA	Recommended Dietary Allowance
BWG	Body Weight Gain
ADG	Average Daily Weight Gain
FI	Feed Intake
FCR	Feed Conversion Ratio
SOD	Superoxide Dismutase
GSH-px	Glutathione Peroxidase
MDA	Malondialdehyde
TNF-α	Tumor Necrosis Factor-α
QIIME	Quantitative Insights Into Microbial Ecology
OTU	Operational Taxonomic Unit
PCoA	Principal Coordinate Analysis

## References

[1] Bai S., Zhang M., Tang S., Li M., Wu R., Wan S., Chen L., Wei X., Feng S. (2024). Effects and Impact of Selenium on Human Health, A Review. Molecules.

[2] Rayman M.P. (2000). The importance of selenium to human health. Lancet.

[3] Zhang F., Li X., Wei Y. (2023). Selenium and Selenoproteins in Health. Biomolecules.

[4] Shahidin, Wang Y., Wu Y., Chen T., Wu X., Yuan W., Zhu Q., Wang X., Zi C. (2025). Selenium and Selenoproteins: Mechanisms, Health Functions, and Emerging Applications. Molecules.

[5] Hsiao Y.F., Huang S.C., Cheng S.B., Hsu C.C., Huang Y.C. (2024). Glutathione and Selenium Supplementation Attenuates Liver Injury in Diethylnitrosamine-Induced Hepatocarcinogenic Mice by Enhancing Glutathione-Related Antioxidant Capacities. Int. J. Mol. Sci..

[6] Varlamova E.G. (2025). Roles of selenium-containing glutathione peroxidases and thioredoxin reductases in the regulation of processes associated with glioblastoma progression. Arch. Biochem. Biophys..

[7] Liu Y., Uyanga V.A., Jiao H., Wang X., Zhao J., Zhou Y., Lin H. (2023). Effects of feeding strategies on eggshell quality of laying hens during late laying period. Poult. Sci..

[8] Yao W., Wang E., Zhou Y., Han Y., Li S., Yin X., Huang X., Huang F. (2023). Effects of garcinol supplementation on the performance, egg quality, and intestinal health of laying hens in the late laying period. Poult. Sci..

[9] Schrauzer G.N. (2000). Selenomethionine: A review of its nutritional significance, metabolism and toxicity. J. Nutr..

[10] Zhao X., Liang K., Zhu H., Wang J. (2021). Health risk assessment of heavy metals contamination in selenium-enriched eggs. Environ. Sci. Pollut. Res. Int..

[11] Wu G., Zhou T., Ma P., Xie B., Li W., Gong S., Xue F. (2023). Mechanism determination on the interactive effects between host immunity and gut microbiome to resist consecutive hydrogen sulfide inhalation of laying hens. Poult. Sci..

[12] Huang J., Xie L., Song A., Zhang C. (2022). Selenium Status and Its Antioxidant Role in Metabolic Diseases. Oxidative Med. Cell. Longev..

[13] (2017). Determination of Selenium in Food.

[14] Pan X., Xue F., Nan X., Tang Z., Wang K., Beckers Y., Jiang L., Xiong B. (2017). Illumina Sequencing Approach to Characterize Thiamine Metabolism Related Bacteria and the Impacts of Thiamine Supplementation on Ruminal Microbiota in Dairy Cows Fed High-Grain Diets. Front. Microbiol..

[15] Fung C., Rusling M., Lampeter T., Love C., Karim A., Bongiorno C., Yuan L.L. (2021). Automation of QIIME2 Metagenomic Analysis Platform. Curr. Protoc..

[16] Aßhauer K.P., Wemheuer B., Daniel R., Meinicke P. (2015). Tax4Fun: Predicting functional profiles from metagenomic 16S rRNA data. Bioinformatics.

[17] Hariharan S., Dharmaraj S. (2020). Selenium and selenoproteins: It’s role in regulation of inflammation. Inflammopharmacology.

[18] He D., Wu H., Jiang H., Zhang Z., Wang C., Wang D., Wei G. (2024). Screening of Selenium/Glutathione-Enriched Candida utilis and Its Anti-inflammatory and Antioxidant Activities in Mice. Biol. Trace Elem. Res..

[19] Zhang T., Li Q., Cheng L., Buch H., Zhang F. (2019). Akkermansia muciniphila is a promising probiotic. Microb. Biotechnol..

[20] Derrien M., Turroni F., Ventura M., van Sinderen D. (2022). Insights into endogenous Bifidobacterium species in the human gut microbiota during adulthood. Trends Microbiol..

[21] Liu L., Chen F., Qin S., Ma J., Li L., Jin T., Zhao R. (2019). Effects of Selenium-Enriched Yeast Improved Aflatoxin B1-Induced Changes in Growth Performance, Antioxidation Capacity, IL-2 and IFN-γ Contents, and Gene Expression in Mice. Biol. Trace Elem. Res..

[22] Arnér E.S.J. (2020). Common modifications of selenocysteine in selenoproteins. Essays Biochem..

[23] Cani P.D., Depommier C., Derrien M., Everard A., de Vos W.M. (2022). Akkermansia muciniphila: Paradigm for next-generation beneficial microorganisms. Nat. Rev. Gastroenterol. Hepatol..

[24] Zhou X., Hu M., Luo J., Xie B., Ma P., Wu G., Xue F. (2023). Resistant effects determination of Lactobacillus supplementation on broilers to consecutive hydrogen sulfide exposure. Poult. Sci..

[25] Baffert C., Kpebe A., Avilan L., Brugna M. (2019). Hydrogenases and H(2) metabolism in sulfate-reducing bacteria of the Desulfovibrio genus. Adv. Microb. Physiol..

[26] Zhou H., Huang D., Sun Z., Chen X. (2024). Effects of intestinal Desulfovibrio bacteria on host health and its potential regulatory strategies: A review. Microbiol. Res..

[27] Baske M.M., Timmerman K.C., Garmo L.G., Freitas M.N., McCollum K.A., Ren T.Y. (2024). Fecal microbiota transplant on Escherichia-Shigella gut composition and its potential role in the treatment of generalized anxiety disorder: A systematic review. J. Affect. Disord..

[28] Zhao J., Bai M., Ning X., Qin Y., Wang Y., Yu Z., Dong R., Zhang Y., Sun S. (2022). Expansion of Escherichia-Shigella in Gut Is Associated with the Onset and Response to Immunosuppressive Therapy of IgA Nephropathy. J. Am. Soc. Nephrol. JASN.

[29] Zelová H., Hošek J. (2013). TNF-α signalling and inflammation: Interactions between old acquaintances. Inflamm. Res..

[30] Yang Z., Min Z., Yu B. (2020). Reactive oxygen species and immune regulation. Int. Rev. Immunol..

